# Realistic and Spherical Head Modeling for EEG Forward Problem Solution: A Comparative Cortex-Based Analysis

**DOI:** 10.1155/2010/972060

**Published:** 2010-02-14

**Authors:** Federica Vatta, Fabio Meneghini, Fabrizio Esposito, Stefano Mininel, Francesco Di Salle

**Affiliations:** ^1^DEEI, University of Trieste, Via A. Valerio 10, 34127 Trieste, Italy; ^2^Department of Neurological Sciences, University of Naples Federico II, II Policlinico Padiglione 17, Via S. Pansini 5, 80131 Naples, Italy; ^3^Department of Cognitive Neuroscience, Maastricht University, P.O. Box 616, 6200 MD Maastricht, The Netherlands

## Abstract

The accuracy of forward models for electroencephalography (EEG) partly depends on head tissues geometry and strongly affects the reliability of the source reconstruction process, but it is not yet clear which brain regions are more sensitive to the choice of different model geometry. In this paper we compare different spherical and realistic head modeling techniques in estimating EEG forward solutions from current dipole sources distributed on a standard cortical space reconstructed from Montreal Neurological Institute (MNI) MRI data. Computer simulations are presented for three different four-shell head models, two with realistic geometry, either surface-based (BEM) or volume-based (FDM), and the corresponding sensor-fitted spherical-shaped model. Point Spread Function (PSF) and Lead Field (LF) cross-correlation analyses were performed for 26 symmetric dipole sources to quantitatively assess models' accuracy in EEG source reconstruction. Realistic geometry turns out to be a relevant factor of improvement, particularly important when considering sources placed in the temporal or in the occipital cortex.

## 1. Introduction

Localization of neural brain sources is important in several areas of research of basic neuroscience, such as cortical organization and integration [1], and in some areas of clinical neuroscience such as preoperative planning [2] and epilepsy [3]. Localization of neural brain sources based on electroencephalography (EEG) uses scalp potential data to infer the location of underlying neural activity [1]. This procedure entails with (i) modeling the brain electrical activity, (ii) modeling the head volume conduction process for linking the neural electrical activity to EEG recordings, and (iii) reconstructing the brain electrical activity from recorded EEG data (measured scalp potentials). The first two modeling steps serve to solve the so-called EEG forward problem, which describes the distribution of electric potentials for given source locations, orientations, and signals; the following step is the inverse of the previous ones, thereby it is commonly referred to as the EEG inverse problem solution.

A model of brain electrical activity (in short “source model”) is composed of bioelectric units distributed within the entire brain volume or over specific brain surfaces or confined to a few brain locations. A single source unit is often modeled as a current dipole, which well approximates the synchronized synaptic currents at a columnar level [4]. When confined to the cerebral cortex, the orientation of the current dipoles can be either free or constrained to be perpendicular to the cortical surface [5].

Linking the source model to the physical electromagnetic signals measurable at the sensor locations on the scalp (forward model) requires constructing a volume conductor model that explains the propagation of the currents throughout the human head in terms of geometry and conductivity of this medium. Modeling errors produced by the differences between the actual head and the volume conductor model affect the accuracy of the EEG forward and hence of the inverse problem solution, as the observed scalp potentials are determined not only by the location and strength of the neural generators but also by the geometry and the conductive properties of the head. Modeling errors include differences in actual head and model shape, skull thickness, and electrical conductivities of the head tissues. This study focuses on the effect of head model geometry on EEG forward solution. Historically, the volume conductor head model assumes that the head consists of a set of three or four concentric homogeneous spherical shells, respectively, representing brain (white and gray matter), cerebrospinal fluid (CSF), skull, and scalp [6]. Sphere-shaped head models are computationally efficient in forward problem formulation and estimation, since they allow using analytical solutions. Of course, they seriously lack in geometrical adherence of the assumed shape with respect to a real human head. The “sensor-fitted sphere” approach fits a multilayer sphere individually to each sensor and has shown to produce some improvement over standard spherical models [7]. More accurate forward solutions become possible by using numerical algorithms, such as the boundary element method (BEM) [8], finite-element method (FEM) [9] and finite difference method (FDM) [10] algorithms. These numerical models allow incorporating the realistic geometry of the head and brain after reconstruction of the anatomical structure from individual or standardized magnetic resonance imaging (MRI) data sets. Previous studies [6–12] have found that a more realistic head model performs better than a less complex, for example, spherical, head model in EEG simulations, since volume currents are more precisely taken into account. More specifically, the BEM approach is able to improve the source reconstruction in comparison with spherical models, particularly in basal brain areas, including the temporal lobe [13], because it gathers a more realistic shape of brain compartments of isotropic and homogeneous conductivities by using closed triangle meshes [14]. The FDM and the FEM allow better accuracy than the BEM because they allow a better representation of the cortical structures, such as sulci and gyri in the brain, in a three-dimensional head model [15]. 

The effect of head model geometry on the EEG forward solution has been considered in several previous studies [12]. These studies analyzed the differences in EEG forward and inverse problem solution due to different spherical or realistic model geometry [16–18], evaluated the effects of variations in the skull thickness [19–21] or due to different model complexity [11, 15], presenting results for particular cases of head models. In [22] the effect of few millimeters random variations in the realistic head shape on the EEG forward and inverse problems was studied. The localization error when solving the inverse problem with head models from several different individuals was studied by [23, 24]. The effect of the head shape variations on the EEG forward and inverse problems was studied in [25] building a random head model based on a set of 30 deterministic models from adults in comparison with a standard average head model. For a dipolar source model, the effect of the head shape variations on the EEG inverse problem due to the random head model resulted slightly larger than the effect of the electronic noise present in the sensors. With the aim of defining a brain that is more representative of the population, the Montreal Neurological Institute (MNI) defined a standard brain by using a large series of MRI scans on normal controls. The current standard MNI template is the ICBM152, which is based on the average of 152 normal MRI scans, thus reflecting average neuroanatomy. In this paper we readdress the effect of realistic geometry in head modeling by adopting the MNI standard anatomy as the most typical real geometry, seeking for more general results also extensible to other application studies in this field, given the above specified characteristics of the MNI template. A realistic highly heterogeneous FDM model of the head based on the MNI anatomy has been developed for these purposes, since an FDM captures complex head geometry and accurately describes the boundary conditions of different tissues with unique conductivity values, including skull orifices [11]. The aim of the presented study is to investigate the accuracy in terms of EEG source modeling that can be achieved adopting realistic, either surface-based (BEM) or volume-based (FDM), or spherical geometries in standard head modeling. We present here a detailed computer simulation study in which the performances in terms of accuracy of three different four-shell head models are compared, the realistic MNI-based BEM and FDM and the sensor-fitted spherical-shaped model. As figures of merit for the comparative analysis, the point spread function (PSF) maps and the lead field (LF) correlation coefficients are used. The models used in the present work are noise-free. Although noise modeling is also important in source localization [2], the purpose of the present study is the punctual evaluation of differences that arise from specific anatomically relevant geometrical modeling of the human brain.

## 2. Material and Methods

**Figure 1:**
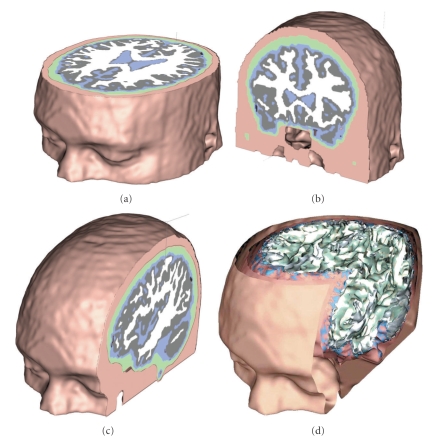
(a), (b), and (c): Realistic FDM model based on the MNI anatomy composed by four compartments representing scalp (pink), skull (green), CSF (blue), and brain, given by fusion of grey matter and white matter. (d): The complete 3D model, with rendered surfaces.

**Figure 2:**
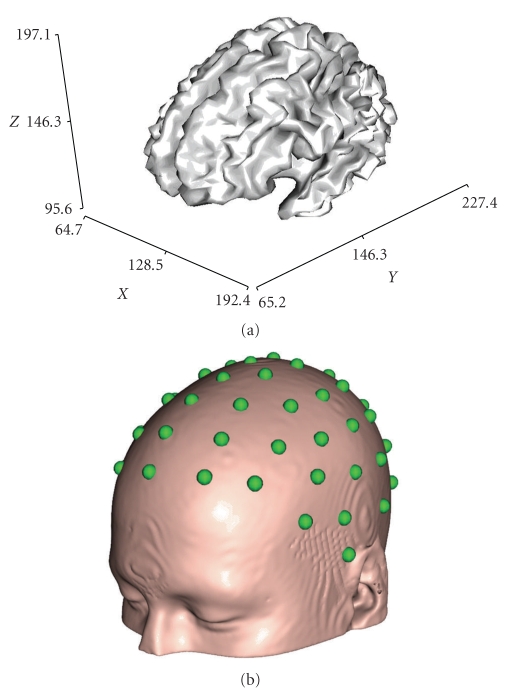
(a): Brain cortex mesh, representing the source space and (b): Scalp surface with spheres indicating the electrodes positions on scalp.

**Figure 3:**
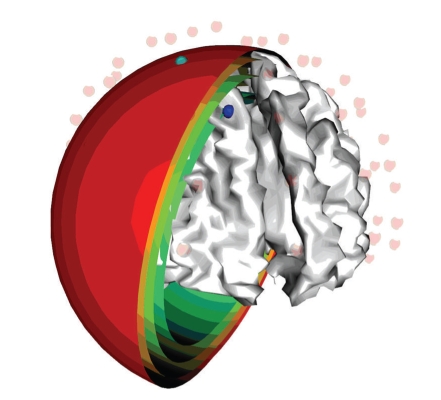
Sensor-fitted 4-shells concentric-spheres model; blue dot: active cortical source; green dot: sensor to which the model is fitted.

A realistic-shaped FDM volume conductor model of the head was derived from an averaged T1-weighted MRI dataset, available from the Montreal Neurologic Institute (http://www.mni.mcgill.ca/). Segmentation by BrainSuite analysis tool (http://brainsuite.usc.edu/) was used to identify the following five tissue types in the head: scalp, skull, cerebrospinal fluid (CSF), gray matter, and white matter (see Figure 1). For the purpose of this comparative study, only four compartments have been set in the model, corresponding to scalp, skull, CSF, and brain (see Figure 1(d)), unifying gray and white matter tissues. In addition to the standard three compartments of scalp, skull, and brain, the CSF layer has been considered as it plays an important role in modifying the scalp potentials and can also influence the inverse source localizations [11]. 62 electrodes positions have been defined evenly spaced over the scalp surface of the realistic head model (see Figure 2). For the comparative study, using the same segmentation results, a four-shell BEM head model has been built [7] with the *BrainStorm* toolkit (http://neuroimage.usc.edu/brainstorm/), after resampling each surface mesh from the original tessellation to 1500 vertices. Finally, a spherical head model has been developed, composed of four concentric spheres representing scalp, skull, CSF, and brain (see Figure 3) with the proportions for the radii of the layers of 1:0.95:0.87:0.84. The “sensor-fitted sphere” approach has been followed [7], according to which the multilayer sphere is fitted individually to each sensor. The conductivity values assigned to the compartments of all the analyzed models were 0.33 S/m for the scalp, 0.0042 S/m for the skull, 1.79 S/m for the CSF, and 0.33 S/m for the brain [26]. The brain cortex mesh was reconstructed from gray matter segmentation and used as space for placing the sources (see Figure 2). The head surface mesh was reconstructed from the unsegmented MNI images and used for placing the 62 EEG electrodes. 

**Figure 4:**
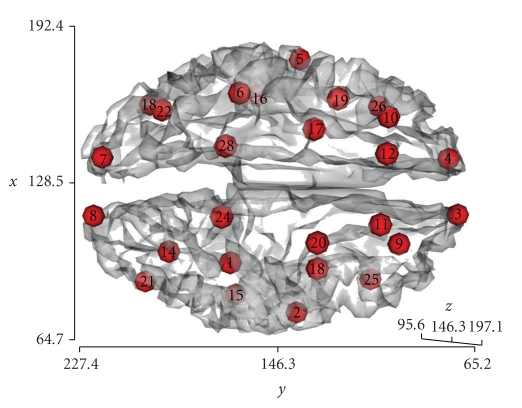
The simulated 26 cortical sources in the Talairach coordinate system.

The sources used for the simulation study are shown in Figure 4. In detail, 5000 evenly spaced points on the brain cortex mesh were initially considered as possible source positions while 26 “true” source positions have been placed in specific vertices of this mesh. The 26 source positions have been selected in order to achieve a rather uniform spatial sampling of the source space, with the aim of investigating the main differences that can be observed in terms of source reconstruction for the various cortical regions in the spherically approximated and in the two different superficial- and volume-based realistic models. For each source position, three single dipole sources have been placed, oriented parallel to the *x*, *y,* or *z* orthogonal Cartesian axes according to the “Talairach” coordinate system, since a source with generic orientation can be always decomposed in its components along the coordinate axes [6]. The study was performed using the numerical FDM for EEG forward problem solution presented in [10], the Galerkin BEM with linear basis algorithm described in [7] for BEM, and analytic calculations for the spherical model [7].

The lead fields of dipoles at the 5000 positions on the cortex mesh were computed and stored for the 62 electrode scalp positions. This procedure has been repeated for each source orientation (*x*, *y*, and *z*) for the same source position, for the realistic BEM and FDM models, and for the sensor-fitted spherical model. Hence, nine leadfield matrices of 62 × 5000 elements were obtained [14], in which each column vector gives the leadfield potentials at the 62 electrodes for each of the 5000 sources in the cortex mesh. Due to the linearity of both the forward and inverse problems, a measure of the estimation error can be obtained by means of the “point spread function” (PSF) [27–29]. The PSF can be calculated, for each source location and orientation, by computing the sensitivity of the estimate at a location *j* to activity at location *i*, after estimating the correlation coefficient between the corresponding column vectors of the leadfield matrices. This procedure, when repeated for each of the 5000 points of the source space in the cortical mesh, leads to the definition of a PSF map for each active source and each orientation; thus, we obtained 78 PSF maps for each model. Given its definition, the PSF function specifies a measure of the spatial blurring of the true activity at any given source position. Therefore, a location with lower PSF is expected to have a smaller spatial extent and higher estimation accuracy. A root mean squared (RMS) superimposition of the effects given by the three source orientations has then been computed, in order to gather a broader vision of the PSF behavior. In order to quantify the differences for the head models considered for each specified source, a measure of the full width at half maximum (FWHM) for the PSF function has also been estimated for each source for both the realistic and the spherical models.

## 3. Results

**Figure 5:**
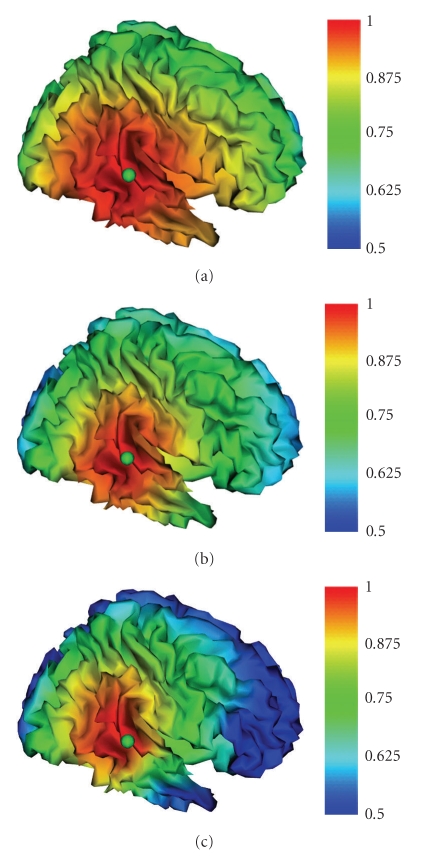
PSF maps obtained for source 2 placed in the temporal region, for the *x*-oriented source, in the Talairach coordinate system: (a) sensor-fitted spherical model; (b) BEM model; (c) FDM model.

The PSF maps on the cortex mesh have been computed for each source location and orientation for a total of 78 PSF maps for the realistic and the spherical head models. The visual inspection of the PSF maps allowed a qualitative evaluation of the spatial blurring of the true activity at the considered source position for the specific head model. The obtained results showed in many cases marked differences between the realistic and the sensor-fitted spherical models when applied to the same source space (cortex) and generally indicated the presence of a more pronounced spatial blurring for the latter model, as evidenced by a broader extent of higher PSF values, with respect to the same source in the realistic BEM and FDM models. Figure 5 shows an example of the results obtained for source 2, placed in the temporal region and *x*-oriented. The PSF maps in the three models indicate the presence of a more pronounced spatial blurring for the sensor-fitted spherical model, evidenced by a broader extent of higher PSF values in the figure, with respect to the same source in the realistic BEM and in the FDM models. 

To quantitatively compare, for the different head models, the spatial characteristics of the PSF maps at any given source position, and hence their power of discrimination for the EEG source reconstruction, the mean and minimum values of the obtained PSFs have been reported and compared for all the 78 analyzed dipole sources, being 1 the maximum PSF value in each condition, for the realistic BEM and FDM and the sensor-fitted spherical models. Tables 1 and 2 summarize the quantitative results of the performed analysis on the PSF maps. A closer inspection of the PSF values presented in Tables 1 and 2 indicates that the reported mean PSF values are larger in the realistic BEM than in the FDM model in 79% of the total tested conditions (62 cases over 78), and specifically in 50%, 88%, and 100% of the analyzed situations for the *x*-, *y*-, and *z*-oriented sources, respectively (i.e., 13, 23, and 26 cases over 26, resp.), rising up to 100% for the RMS superimposition of the effects given by the three source orientations. The minimum PSF values result larger in the BEM with respect to the FDM model in 27% of the tested conditions (21 cases over 78), in the 0%, 4%, and 77% of cases for the *x*-, *y*-, and *z*-oriented sources, respectively, rising up to 85% for the RMS data. The spherical head model (SPH) presents larger mean PSF values with respect to both the realistic BEM, and FDM models, for 60% (BEM) and 92% (FDM) of the total tested conditions (47 and 72 cases over 78, resp.), with minimum PSF values larger in 97% and 85% of the total conditions (76 and 66 cases over 78, resp.). The analysis of the RMS superimposition of the effects given by the three source orientations indicates that the spherical model shows larger mean PSF values in 85% of the tested conditions with respect to the BEM model, rising up to 96% for the FDM; the minimum PSF values result larger in 96% of the tested conditions for the BEM model and in 54% for the FDM. For *x*-oriented sources the spherical model shows larger mean and minimum PSF values in 96% and 100% of the tested conditions, respectively, for both the BEM and the FDM models. The *y*-oriented sources show a similar behavior with larger mean and minimum PSF values for the spherical model in 85% and 100% of the tested conditions with respect to BEM, and in 92% and 54% with respect to FDM. For *z*-oriented sources, the minimum PSF values result larger for the spherical model in 92% (BEM) and 100% (FDM) of the tested conditions. Conversely, the *z*-oriented sources present smaller mean PSF values for the spherical model in comparison with the BEM in all the 26 tested conditions, while for the FDM this situation shows up only for 3 cases out of 26, thus giving larger mean PSF values for the spherical model with respect to FDM in 88% of the tested conditions. The evaluation of the mean ± SD values of the reported mean PSF values for the three models analyzed, listed in Table 1, confirmed these trends for the three source orientations and for the RMS data. The data from all the analyzed samples (FDM, BEM and SPH) resulted normally distributed and nine two-tailed paired *t*-tests have been performed to investigate differences between the spherical and the realistic models, that is, FDM versus BEM, FDM versus SPH and BEM versus SPH for the three source orientations. Statistically significant differences have been found in the mean PSF values in 7 cases out of the total 9: for all source orientations for both BEM versus SPH (*x* : *p* = 6.43 × 10^−7^; *y* : *p* = 1.90 × 10^−5^; *z* : *p* = 9.45 × 10^−8^) and FDM versus SPH (*x* : *p* = 1.25 × 10^−10^; *y* : *p* = 6.74 × 10^−8^; *z* : *p* = 3.13 × 10^−4^) and for the *z*-oriented sources (*p* = 3.23 × 10^−13^) in FDM versus BEM. No statistically significant differences have been found in 2 cases, that is, for the *x*-oriented (*p* = 0.70) and for the *y*-oriented sources (*p* = 0.28) in FDM versus BEM. The analysis on the minimum PSF data led to similar results, with 7 cases of significant differences out of the total 9: for all source orientations in BEM versus SPH (*x* : *p* = 1.43 × 10^−13^; *y* : *p* = 9.55 × 10^−11^; *z* : *p* = 2.17 × 10^−8^), for the *x*-oriented (*p* = 2.35 × 10^−6^) and the *z*-oriented sources (*p* = 2.02 × 10^−9^) in FDM versus SPH (*y*-oriented sources: *p* = 0.29), and for the *x*-oriented (*p* = 1.49 × 10^−12^) and the *y*-oriented sources (*p* = 3.49 × 10^−9^) in FDM versus BEM (*z*-oriented sources: *p* = 0.08). The RMS data showed significant differences in both FDM versus BEM and FDM versus SPH for either the mean (FDM versus BEM: *p* = 1.90 × 10^−10^); FDM versus SPH: *p* = 1.90 × 10^−11^) and the minimum PSF values (FDM versus BEM: *p* = 8.34 × 10^−5^); FDM versus SPH: *p* = 4.71 × 10^−7^).

**Figure 6:**
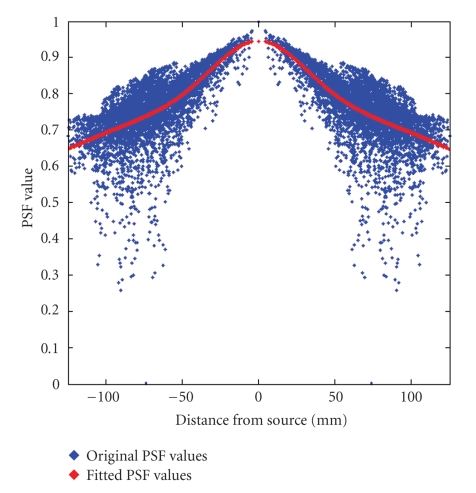
Example of PSF values distribution versus distance from source, and fitting with biexponential Gaussian curve.

**Figure 7:**
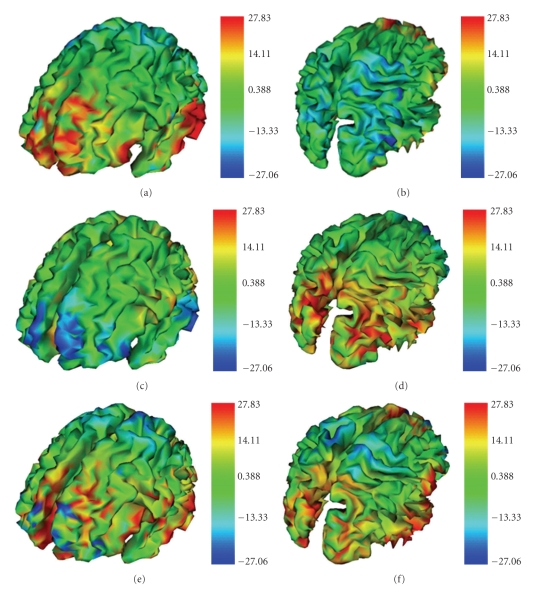
Differences between the FWHM values of the RMS PSF maps for the three couples of analyzed head models, computed as the difference between values of FWHM_Model1_ andFWHM_Model2_ over the cortex mesh (FWHM_Model1_ − FWHM_Model2_). (A, B) FWHM_BEM_ − FWHM_FDM_; (C, D) FWHM_SPH_ − FWHM_BEM_; (E, F) FWHM_SPH_ − FWHM_FDM_. Positive values in the computed FWHM_Model1_ − FWHM_Model2_ differences are represented in red; negative values are represented in blue. Red zones correspond to a smaller spatial extent and hence to a better capacity in terms of spatial discrimination of neural sources of Model 2 with respect to Model 1 for the different model pairs.

Following spatial smoothing, the quantitative evaluation of the PSF maps has been conducted by plotting, for each of the 78 analyzed sources, the obtained PSF values as function of the distance from the source position and fitting the map values with the corresponding best-fitting Gaussian-like function (biexponential Gaussian), as shown in Figure 6. The spatial extent of the PSF function, measured in mm, has been quantified by means of its full width at half maximum (FWHM) measure. The obtained PSF FWHMs have been reported and compared for all the 26 analyzed dipole sources for each source orientation, for the realistic BEM and FDM and the sensor-fitted spherical models. Table 3 summarizes the quantitative results of the performed analysis on the PSF maps. Basing upon a closer inspection of the PSF FWHM results presented in Table 3, it can be observed that the realistic FDM model presents an improvement over BEM in 68% of the total tested conditions (53 cases over 78), and specifically in 54% of the *x*-oriented sources (14 cases over 26), in 81% and 69% for the *y*- and *z*-oriented sources, respectively (21 and 18 cases over 26, resp.), and in 38% of the RMS (10 over 26). The realistic BEM presents an improvement over the spherical model in 62% of the total tested conditions (48 cases over 78), in 77%, 73%, 35%, and 77% of the situations for the *x*-, *y*-, and *z*-oriented sources and RMS, respectively. The improvement of FDM over the spherical model shows up in 88% of the analyzed situations for all the three source orientations, and in the 66% for the RMS. These trends are also confirmed by the mean ± SD values of the reported PSF FWHM results for the three models, shown in Table 3. Nine two-tailed paired *t*-tests have been performed to investigate differences between the spherical and the realistic models (pairs FDM versus BEM, FDM versus SPH, and BEM versus SPH) for the three source orientations. Statistically significant differences have been found in 7 out of the total 9 cases analyzed: for all source orientations in FDM versus SPH (*x* : *p* = 2.03 × 10^−6^; *y* : *p* = 1.98 × 10^−4^; *z* : *p* = 1.93 × 10^−3^), for the *y*- and *z*-oriented sources in FDM versus BEM (*y* : p = 1.69 × 10^−3^; *z* : *p* = 3.66 × 10^−4^), and for the *x*- and *y*-oriented sources in BEM versus SPH (*x* : *p* = 1.67 × 10^−2^;* y* : *p* = 2.83 × 10^−2^). The two-tailed paired *t*-tests performed on the RMS results showed significant differences in the FDM versus SPH pair (*p* = 1.36 × 10^−2^) and nonsignificant differences in the FDM versus BEM (*p* = 0.91) and in the BEM versus SPH (*p* = 5.63 × 10^−2^). In order to gather a broader evaluation of the PSF behavior on the overall brain cortex, we extended the evaluation of the FWHM PSF to all the 5000 points of the cortex surface. Figure 7 shows the differences between the FWHM RMS PSF maps between couples of different head models, to investigate the principal benefits or pitfalls given by the adoption of the different head models.

## 4. Discussion

The dissimilarities between the forward fields simulated for the spherically approximated and the two different superficial- and volume-based realistic models have been investigated on a standard real cortex geometry by means of analysis of the lead fields. The Point Spread Function (PSF) has then been used to quantify the amount of spatial blurring of simulated cortical activity effects. The reported PSF values generally indicate a smaller extent, and hence a clear improvement, for the FDM realistic model in comparison with the BEM, and of the BEM model in comparison with the sensor-fitted spherical model (see Figures 5 and 7 and Tables 1–3). This can be better observed analyzing the mean ± SD values of the reported PSF FWHM results for the three models, for which a clear trend in this sense can be observed for the *x*- and *y*-oriented sources, a slight worsening can be observed for the *z*-oriented sources in BEM versus SPH accompanied by an improvement in both FDM versus BEM and FDM versus SPH, and a slight improvement in FDM versus BEM for the RMS data, accompanied by an improvement of both FDM and BEM versus SPH. This trend in FDM versus BEM is reported also by the mean PSF values that are larger in the realistic BEM than in the FDM model in most of the total tested conditions for the separate source orientations, rising up to totality for the RMS superimposition of the effects given by the three source orientations. This situation is accompanied by generally lower minimum PSF values for the BEM with the three separate source orientations but not for the RMS data, leading in general to smaller PSF FWHMs for FDM versus BEM with the separate source orientations, inferring a lower spatial blurring effect for FDM with respect to BEM; for the RMS superimposition of the effects given by the three source orientations the PSF FWHMs result rather similar, as indicated also by the presence of statistical significant differences in FDM versus BEM for the only *y*- and *z*-oriented sources. The resulting trend in SPH versus BEM and versus FDM is also confirmed by the larger mean and minimum PSF values presented by the spherical head model with respect to both the realistic BEM and FDM in most of the total tested conditions for the separate three source orientations and for the RMS superimposition of the effects, with the exception of smaller mean PSF values for the spherical model than for the BEM for the *z*-oriented sources. The exception behavior observed for the *z*-oriented sources is reflected also by their PSF FWHMs, with an improvement of the realistic BEM over the spherical model in only 35% of the tested situations and by the presence of statistical significant differences for all source orientations and for the RMS values in the pair FDM versus SPH and for only the *x*- and *y*-oriented sources in the pair BEM versus SPH. It should be however observed that the improvement of one of the models with respect to the other one might be evaluated not only in terms of the sole mean PSF or of the PSF FWHM value but also in terms of the combined information which can be gathered basing upon these data. The relationship between the PSF FWHM and the standard deviation **σ**of the PSF can be in fact expressed as FWHM = 2√2ln 2*σ* ≈ 2.35482*σ*. Considering that the signal-to-noise ratio (SNR) of the PSF can be expressed as the reciprocal of the coefficient of variation (CV) of the PSF distribution, which can be in turn expressed as the ratio of the standard deviation **σ** and the mean PSF, the SNRs of the PSF distributions for the BEM and the SPH models can be computed based upon the mean PSFs and the standard deviations obtained by the PSF FHWM values reported in Tables 1 and 3. The evaluation of the SNRs of the *z*-oriented sources for the BEM and the SPH models indicates that there is a general increase (22.7% mean) in the SNR for the BEM model with respect to the spherical one for all the *z*-oriented tested sources. 

A worsening of both the realistic models versus the spherical can be observed for sources in the frontal lobe (Figures 7(c)–7(e), sources 3-4 in Figure 4), positioned in proximity of the frontal sinus. This might be due to the vicinity of the paranasal sinuses, which are actually filled with humid air but are nonetheless modeled as compact bone in our realistic models, in order not to introduce a fifth compartment. To test this hypothesis, sources 9-10 and 25-26 (see Figure 4) have been selected on the cortex mesh, placed laterally to sources 3 and 4 and to the paranasal sinuses, with the positive effect of improvement in terms of spatial blurring given by the realistic model (see Figures 7(c)–7(e)).

Results for sources placed in the temporal cortex (namely 2–5, 15-16, 17-18, and 19-20 in Figure 4) indicate that the realistic model generally leads to an improvement in terms of spatial blurring with respect to spherical model. The same trend is presented by realistic FDM with respect to BEM. These results are in agreement with previous studies that showed that a 3-compartment realistic BEM model of the head outperformed a 3-shell spherical model, particularly in the temporal lobe [13]. This trend is also confirmed for sources which are positioned in the occipital cortex, namely, 7-8, 13-14, 21-22, and 23-24, again demonstrating that the adoption of a realistic model instead of a spherical one can lead to benefits in terms of power of discrimination for the reconstruction of these sources. The spherical model results in fact to perform best in the more spherical upper parts of the brain (see Figures 7(c)–7(f)), but fails in the temporal and occipital lobe areas, which cannot be well represented by the spherical shells. These findings confirm earlier studies that showed similar behavior [24]. Moreover, for sources located in parieto-occipital areas (see Table 3 for sources n. 13–17 and 21–24 and see Figures 7(b), 7(d), 7(e)), PSF parameters exhibit smaller FWHM for the realistic model, compared to the spherical one, with slightly smaller FWHM for BEM with respect to FDM that might be due to the smoothing of sulci presented by BEM. 

The computational performances of the spherically approximated and of the two different BEM and FDM realistic models analyzed can provide also useful elements in order to assess cost-benefit of the specific model adopted. Computational performance was determined for the spherical and the BEM models with a standard PC (AMD64 3.00 GHz/3 GB RAM, 2 MB cache 2L) and for the FDM model with a Linux cluster PC composed by 8 elements of the same type (i.e., the above described unit as the front-end node plus 7 AMD64 3.00 GHz / 2 GB RAM elements), as the FDM EEG forward problem solution was set up on a parallel computing implementation, given the higher computational load presented by the volume-based realistic models (FDM and FEM) [12]. When measuring the wall-clock time, it should be distinguished between the setup-computation that only has to be carried out once per head model for the building of the lead field matrix and the forward computations that have to be carried out hundreds or hundreds of thousands of times depending on the inverse problem solution procedure [30]. During the setup, the computation of the leadfield matrix by means of the FDM solver took about 5.7 hours, that is, about 330 seconds per sensor. The resulting linear system matrix for the computation of each column vector of the lead field matrix had a size of about 14 GB, while the final lead field matrix had a size of about 8 MB for all the three models considered. The computation of the leadfield matrix by means of the BEM solver took about 4.1 hours, being this the total time needed for the transfer matrix setup and decomposition with additional 12 s for the computation of the columns of the leadfield matrix for all the sensors. The computation of the leadfield matrix by means of the adopted sensor-fitted spherical approach needed a time of 0.82 hours (2960  seconds). It should finally be underlined that the cost-benefit of having selected one or the other of the analyzed models should consider only the initial setup time for computing and storing the leadfield matrixes for the different models [30]. The choice of adopting one specific head model has then to be made in terms of costs basing on the one-time initial setup time, and taking into consideration for the benefits the factors of improvement that are gathered by the different models which have been here evaluated in terms of the specific PSF maps.

In conclusion, the obtained results demonstrate that realistic geometry can provide a factor of improvement which is particularly important when considering sources placed in the temporal or in the occipital cortex. In these situations, using a realistic head model will allow a better spatial discrimination of neural sources in comparison with the spherical model, as it can be appreciated by the analysis of the PSF maps presented in this paper. It is also worth stressing that the results presented in this paper, thanks to the adoption of the MNI-based models, based on a large series of MRI scans on normal controls and thus reflecting average neuroanatomy more representative of the population, can be an enrichment with respect to other studies for the possibility of gathering more general information also extensible to other application studies in this field. 

## Figures and Tables

**Table 1:** Summary of the mean PSF values for the realistic (BEM and FDM) and the sensor-fitted spherical (SPH) models, for sources 1÷26 parallel to *x*, *y*, and *z* orientations and for the RMS maps.

	Mean PSF											
	*x*			*y*			*z*			RMS		
	FDM	BEM	SPH	FDM	BEM	SPH	FDM	BEM	SPH	FDM	BEM	SPH
1	0.76	0.74	0.82	0.74	0.76	0.83	0.65	0.75	0.71	0.71	0.75	0.79
2	0.66	0.67	0.75	0.61	0.64	0.78	0.52	0.77	0.65	0.58	0.70	0.73
3	0.57	0.66	0.69	0.55	0.57	0.46	0.46	0.80	0.49	0.48	0.69	0.56
4	0.51	0.61	0.46	0.55	0.56	0.44	0.52	0.78	0.35	0.47	0.66	0.42
5	0.67	0.71	0.77	0.64	0.71	0.8	0.55	0.80	0.65	0.60	0.74	0.74
6	0.75	0.74	0.8	0.74	0.74	0.82	0.64	0.77	0.7	0.70	0.75	0.78
7	0.6	0.57	0.66	0.58	0.63	0.62	0.52	0.69	0.44	0.56	0.64	0.58
8	0.61	0.56	0.74	0.55	0.62	0.68	0.56	0.70	0.53	0.57	0.63	0.66
9	0.7	0.73	0.8	0.65	0.65	0.81	0.61	0.78	0.68	0.64	0.72	0.77
10	0.72	0.71	0.81	0.66	0.69	0.81	0.59	0.81	0.7	0.64	0.74	0.78
11	0.77	0.75	0.8	0.75	0.76	0.83	0.65	0.79	0.69	0.71	0.77	0.78
12	0.76	0.73	0.79	0.72	0.74	0.81	0.6	0.81	0.66	0.70	0.76	0.76
13	0.74	0.72	0.83	0.69	0.72	0.83	0.66	0.81	0.73	0.69	0.75	0.80
14	0.75	0.69	0.83	0.7	0.72	0.83	0.66	0.78	0.73	0.69	0.74	0.80
15	0.76	0.72	0.82	0.73	0.74	0.84	0.66	0.78	0.72	0.70	0.75	0.80
16	0.76	0.76	0.83	0.74	0.77	0.85	0.66	0.83	0.73	0.70	0.79	0.81
17	0.63	0.72	0.77	0.7	0.54	0.83	0.47	0.81	0.63	0.58	0.70	0.75
18	0.68	0.74	0.78	0.7	0.57	0.83	0.53	0.81	0.67	0.62	0.72	0.76
19	0.74	0.74	0.82	0.73	0.75	0.83	0.66	0.82	0.71	0.70	0.77	0.79
20	0.74	0.75	0.8	0.72	0.71	0.82	0.64	0.74	0.68	0.69	0.73	0.77
21	0.72	0.68	0.8	0.65	0.70	0.82	0.63	0.80	0.68	0.65	0.73	0.77
22	0.72	0.72	0.82	0.68	0.70	0.83	0.63	0.80	0.69	0.66	0.75	0.79
23	0.75	0.72	0.83	0.71	0.74	0.84	0.65	0.79	0.74	0.68	0.76	0.80
24	0.73	0.66	0.82	0.73	0.74	0.83	0.63	0.77	0.73	0.68	0.73	0.80
25	0.64	0.70	0.77	0.64	0.64	0.76	0.57	0.70	0.62	0.59	0.68	0.73
26	0.6	0.64	0.72	0.59	0.65	0.64	0.52	0.77	0.55	0.55	0.69	0.64

Mean	0.69	0.70	0.77	0.67	0.68	0.77	0.59	0.78	0.65	0.64	0.72	0.74
± SD	± 0.07	± 0.05	± 0.08	± 0.07	± 0.07	± 0.11	± 0.06	± 0.04	± 0.10	± 0.07	± 0.04	± 0.09

**Table 2:** Summary of the minimum PSF values for the realistic (BEM and FDM) and the sensor-fitted spherical (SPH) models, for sources 1*÷*26 parallel to *x*, *y* and *z* orientations and for the RMS maps.

	Min PSF											
	*x*			*y*			*z*			RMS		
	FDM	BEM	SPH	FDM	BEM	SPH	FDM	BEM	SPH	FDM	BEM	SPH
1	0.4	0.12	0.42	0.38	0.13	0.28	−0.16	0.10	0.2	0.34	0.38	0.32
2	0.26	0.06	0.3	0.23	−0.03	0.29	0	0.06	0.17	0.25	0.33	0.27
3	0.23	−0.03	0.31	0.11	−0.33	0.06	−0.24	−0.22	−0.07	0.15	0.37	0.18
4	0.21	0.10	0.23	0.11	−0.17	0.08	−0.26	−0.38	−0.11	0.16	0.37	0.16
5	0.27	−0.24	0.3	0.23	0.11	0.16	−0.26	−0.21	0.14	0.27	0.44	0.27
6	0.37	0.09	0.39	0.37	0.06	0.24	−0.28	−0.03	0.18	0.36	0.33	0.30
7	0.23	−0.08	0.24	0.17	−0.21	−0.02	−0.12	−0.10	−0.11	0.21	0.25	0.15
8	0.23	0.08	0.28	0.14	−0.15	0.06	−0.14	−0.06	−0.07	0.23	0.22	0.17
9	0.32	−0.06	0.42	0.09	−0.21	0.38	0.02	−0.09	0.09	0.28	0.38	0.33
10	0.36	0.16	0.43	0.11	0.10	0.38	−0.22	−0.20	0.13	0.25	0.40	0.34
11	0.38	0.08	0.46	0.32	0.08	0.39	−0.09	0.07	0.14	0.31	0.43	0.37
12	0.44	0.12	0.44	0.26	0.14	0.38	−0.32	−0.06	0.13	0.28	0.37	0.35
13	0.31	−0.09	0.37	0.23	−0.15	0.22	−0.16	−0.10	0.13	0.29	0.32	0.27
14	0.33	0.12	0.38	0.23	−0.05	0.23	−0.06	0.05	0.15	0.31	0.30	0.27
15	0.35	0.12	0.39	0.31	0.10	0.26	−0.01	0.10	0.19	0.34	0.37	0.30
16	0.38	−0.03	0.38	0.33	0.00	0.26	−0.4	−0.11	0.19	0.36	0.37	0.31
17	0.31	0.06	0.32	0.27	−0.14	0.28	−0.11	−0.31	0.18	0.31	0.42	0.29
18	0.32	−0.08	0.34	0.33	−0.32	0.3	−0.02	−0.03	0.22	0.28	0.41	0.30
19	0.4	0.11	0.39	0.31	0.17	0.36	−0.28	−0.11	0.18	0.33	0.39	0.34
20	0.34	0.02	0.4	0.29	−0.03	0.36	0.02	0.11	0.14	0.32	0.37	0.35
21	0.29	0.14	0.34	0.08	−0.09	0.28	−0.06	−0.05	0.13	0.29	0.28	0.27
22	0.3	−0.23	0.35	0.15	−0.26	0.19	−0.08	−0.21	0.12	0.28	0.37	0.28
23	0.39	0.06	0.39	0.23	−0.07	0.22	−0.26	−0.04	0.16	0.34	0.29	0.27
24	0.34	0.20	0.39	0.32	−0.02	0.18	−0.17	0.04	0.15	0.33	0.25	0.26
25	0.24	−0.12	0.39	0.11	−0.18	0.34	−0.01	−0.40	0.05	0.25	0.35	0.30
26	0.27	0.17	0.35	−0.05	0.14	0.23	−0.33	−0.31	0	0.21	0.37	0.24

Mean	0.32	0.03	0.36	0.22	−0.05	0.25	−0.15	−0.10	0.11	0.28	0.35	0.28
± SD	± 0.06	± 0.12	± 0.06	± 0.11	± 0.15	± 0.11	± 0.12	± 0.15	± 0.10	± 0.06	± 0.06	± 0.06

**Table 3:** Summary of the FWHM PSF values for the realistic (BEM and FDM) and the sensor-fitted spherical (SPH) models, for sources 1*÷*26 parallel to x, y and z orientations and for the RMS maps.

	FWHM											
	*x*			*y*			*z*			RMS		
	FDM	BEM	SPH	FDM	BEM	SPH	FDM	BEM	SPH	FDM	BEM	SPH
1	66.3	57.7	75.7	55	73.7	80	73.1	61.8	71.6	72.23	61.38	68.81
2	68.2	54.1	81.1	59	76.8	82.4	61.2	81.1	80.2	62.20	54.62	80.19
3	65	97.6	97.6	77.2	70.3	54.8	92.5	114.4	73.9	69.90	100.41	66.67
4	60.1	87.5	41.9	81.5	72.1	40.4	105.7	109.3	54.5	72.49	78.09	46.02
5	63	77.5	78.4	58.7	77.6	79.4	57.3	83.0	73.8	57.89	78.81	77.27
6	70.4	59.9	70.3	59.5	78.7	69.9	61.6	54.1	73.3	65.92	62.26	67.84
7	67.4	57.4	71.3	75.4	91.5	79.6	60.3	63.6	63.4	69.29	65.59	68.98
8	68.5	61.9	95.3	68.8	90.5	85.8	66.7	60.0	86.2	84.33	63.71	77.90
9	71.8	89.0	77.7	83.2	85.3	99.6	64.1	76.8	87.2	85.64	71.69	80.72
10	68.3	86.6	80.7	72.5	84.0	96.1	67.5	90.3	80.1	83.16	73.22	82.23
11	70.5	71.4	64.5	75.2	78.5	88.5	66.9	82.4	75.7	77.09	69.65	69.81
12	65	79.6	68.6	75.3	79.3	91.3	61.8	60.9	76.8	77.88	71.24	66.83
13	72.2	49.6	90.6	66.9	76.0	100.4	63.2	90.3	86.9	68.47	86.60	94.13
14	74.1	58.9	89.5	66.1	83.2	99.0	68.3	59.4	85.6	81.90	61.44	91.65
15	72.1	58.4	82.5	63	78.3	87.1	70.5	63.1	77.8	78.16	61.56	81.94
16	67.8	62.0	76.5	60.2	78.4	77.5	58.1	77.6	73.2	75.18	63.60	76.06
17	50.9	89.3	78.9	62.7	33.7	68.5	63	84.0	74.2	51.55	82.72	67.74
18	60.2	74.4	75.1	58.4	70.1	79.3	58.2	77.6	73.7	56.99	74.41	75.06
19	58.3	67.2	79.4	70.3	75.9	82.1	60.4	78.8	74.3	63.52	77.17	78.88
20	64.6	78.8	80	71.2	77.1	81.6	65.8	78.5	76.2	76.17	72.64	79.15
21	73.7	64.0	89.7	64.1	86.2	98.7	68.7	89.9	83.4	68.56	60.98	88.92
22	71.8	52.5	87.6	66.8	85.6	95.2	58.4	87.7	82	67.03	85.39	87.55
23	71.1	58.8	85.6	65.9	80.1	95.6	69.6	86.1	84	79.21	63.48	89.28
24	69.2	79.1	88.2	71.4	81.9	98.3	66.1	58.2	84.6	69.02	63.07	88.12
25	69.1	92.1	96.3	79.5	66.8	81.5	71.2	69.4	81	69.41	73.55	93.01
26	62.0	79.0	89.3	68.1	66.1	63.7	60.4	97.2	76.5	61.55	76.10	73.57

Mean	67.0	70.9	80.5	68.3	76.8	82.9	66.9	78.3	77.3	71.0	71.3	77.6
± SD	± 5.4	± 14.0	± 11.7	± 7.7	± 10.9	± 14.6	± 10.6	± 15.6	± 7.4	± 8.8	± 10.3	± 10.8
